# Effect of Local Vibration Therapy on Pain, Joint Position Sense, Kinesiophobia, and Disability in Cervical Disc Herniation: A Randomized Controlled Trial

**DOI:** 10.3390/jcm13154566

**Published:** 2024-08-05

**Authors:** Merve Yilmaz Menek, Emre Dansuk, Umut Islam Tayboga

**Affiliations:** Department of Physiotherapy and Rehabilitation, Faculty of Health Sciences, Istanbul Medipol University, 34810 Istanbul, Turkey; edansuk@medipol.edu.tr (E.D.); umut.tayboga@medipol.edu.tr (U.I.T.)

**Keywords:** cervical disc herniation, local vibration therapy, joint position sense, kinesiophobia, neck disability, percussion massage

## Abstract

**Background/Objectives:** Vibration therapy approaches are an effective and safe treatment option for musculoskeletal disorders. This study examines the effects of vibration therapy using a percussion massage gun (PMG) on joint position sense, range of motion, pain, functionality, and kinesiophobia in individuals with cervical disc herniation (CDH). **Methods:** This single-blind randomized controlled trial involved 44 CDH patients divided into a Vibration Group (VG) and a Conventional Group (CG). The CG underwent a standard physiotherapy treatment heat application, Transcutaneous Electrical Nerve Stimulation (TENS), and exercises for range of motion and strengthening. VG received conventional therapy augmented with vibration therapy (VT) via a PMG. Joint position sense (JPS) using the Laser Pointer Assisted Angle Repetition Test; pain intensity with the Visual Analog Scale, kinesiophobia with the Tampa Scale for Kinesiophobia, and cervical dysfunction with the Neck Disability Index were assessed. **Results:** Both groups showed statistically significant improvements in pain, kinesiophobia, disability, and proprioception after treatment (*p* < 0.05). When comparing the difference values between groups, the VG was found to be more effective than the CG in the parameters of VAS activity (*p* = 0.013). The CG had more improvement in JPS neck left rotation than the VG (*p* = 0.000). **Conclusions:** VT, when combined with conventional physiotherapy, is effective in improving pain, proprioception, and functionality in individuals with CDH. These findings support the inclusion of VT as a beneficial adjunct therapy. Further research with larger sample sizes and longer follow-ups is recommended to validate these results and explore the long-term effects of VT on CDH.

## 1. Introduction

Cervical disc herniation (CDH) is characterized by the prolapse of nucleus pulposus material through the annulus into the spinal canal. The local mechanical or chemical irritation of neuronal structures typically results in symptoms of radiculopathy, cervical headache, or myelopathy. While pain is frequently the initial complaint of patients with CDH, depending on the location and degree of the prolapse, symptoms may include postural abnormalities affecting the whole body, as well as fear of falling and movement [1]. According to current research, one of the most common difficulties in neck pain sufferers is a lack of cervical proprioception, which leads to cervical sensorimotor control disorders [2,3].

The physiotherapy and rehabilitation program is usually prescribed after a short period of rest and immobilization. Modalities include a range of motion exercises, strengthening exercises, ice, heat, ultrasound, and electrical stimulation therapy [4].

Recently, vibration therapy (VT) has been widely used in gyms, sports medicine, and rehabilitation clinics with potential beneficial effects to improve muscle strength, balance, bone health, or pain [5]. VT is used to promote edema absorption, increase blood flow, facilitate wound healing, and provide anti-inflammatory and anti-fibrotic properties [6,7]. Furthermore, the effects of VT on pain alleviation have been extensively documented. This approach is beneficial for patients with fibromyalgia, acute and chronic musculoskeletal pain, delayed onset muscle soreness, and myotendinous injuries involving myofascial trigger points [8]. Studies have shown that VT improves pain, function, and sensorimotor function in people with neck pain, and it is recommended to be applied to these individuals [9,10]. According to Beinert et al., vibration increases the afferent input from primary muscle spindle ends, which may lead to an increased reliance on vestibular or cutaneous information by the central nervous system. Patients with neck pain may benefit from this input because they may be able to control erratic afferent input from muscle spindles [11]. Similarly, Wannaprom et al. demonstrated that VT may change cervical proprioception and/or vestibular input in the healthy group [12].

Vibration is used to deliver mechanical vibration, with energy coming from the device directly into the tendon or muscle or indirectly through the hands—when holding a device—or the feet—when standing on a platform [13]. These delivery methods can include handheld vibrating devices, whole-body vibration treatment, local vibration therapy, oscillation vibration, and cycloidal vibration. Up until now, reviews have used the general phrase “vibration therapy” to refer to a variety of devices and procedures [14]. Percussion massage guns are a type of VT that has been used for therapeutic purposes for the last few years. Different manufacturers (e.g., Theragun, Hyperice, Compex) provide vibration devices for both self-massage and massage by a therapist. Such devices are capable of vibrating at different frequencies up to 53 Hz. Depending on the tissue (i.e., soft tissue versus bony tissue), various attachment heads can be fixed to the devices [15]. Studies conducted by Szymczyk et al., Liu et al., and Nakamura et al. have reported that local VT relaxes the fascia and increases the range of motion [16,17,18]. Another study found that local VT induces changes at the tissue level, resulting in an acute increase in the range of motion [19]. Similarly, it has been shown to be more effective than dynamic stretching in enhancing flexibility [20]. Additionally, another study indicated that local VT and static stretching have similar effects on increasing flexibility [21]. Furthermore, one study showed that local VT did not affect delayed onset muscle soreness [22]. There are some studies evaluating various forms of vibration on Delayed Onset Muscle Soreness (DOMS). Recently, whole-body vibration has also been evaluated as a post-exercise recovery treatment to reduce muscle pain and soreness after exercise. Vibration has been demonstrated to improve muscle perfusion and improve lymph flow [23]. Also, the local application of vibration is another form of stimulus propagation that causes smaller changes in the skeletal system. Locally applied vibration contributes to the development of neuromuscular adaptations after both single and multiple uses [24].

The literature on local VT remains inconclusive and contentious. Despite the increasing use of mechanical percussion devices in clinical settings, there is still limited information to effectively guide practitioners [25,26]. A review of the literature reveals that most studies primarily focus on lower extremity issues. There is a noticeable lack of research on local VT targeting the neck region, underscoring the need for further investigation in this area. VT could be potentially harmful to the soft tissue organs within the head in neck region application and therefore the selection of the biomechanical parameters should be well-considered [27]. Due to the human anatomy, vibration transmission occurs segmentally from the vibration source to the feet, then to the calves, up to the thighs, and from the trunk through the neck to the head [28]. The studies recommend a vibration frequency of 30 Hz to reduce the risk of injury to structures of the head [29].

As VT is known to be an essential determinant of neuromuscular responses, it is crucial in the treatment of cervical disc herniation. However, a study investigating the effects of vibration on CDH for this purpose was not found in the literature as we know. This study aimed to investigate the effects of local vibration applied with a percussion massage gun on a joint position sense, pain, kinesiophobia, and functionality in individuals with cervical disc herniation.

## 2. Methods

### 2.1. Study Design and Participants

This study was a randomized, controlled, single-blind trial with participants allocated (1:1) to one of two groups. This trial was approved by the Non-interventional Ethics Committee at Istanbul Medipol University, (E-10840098-772.02-1701, Number: 226). The protocol of the study was registered at ClinicalTrials.gov (NCT06139263). Participants were fully informed about the objectives and extent of the research, and their consent was documented through signed consent forms.

**Figure 1:**
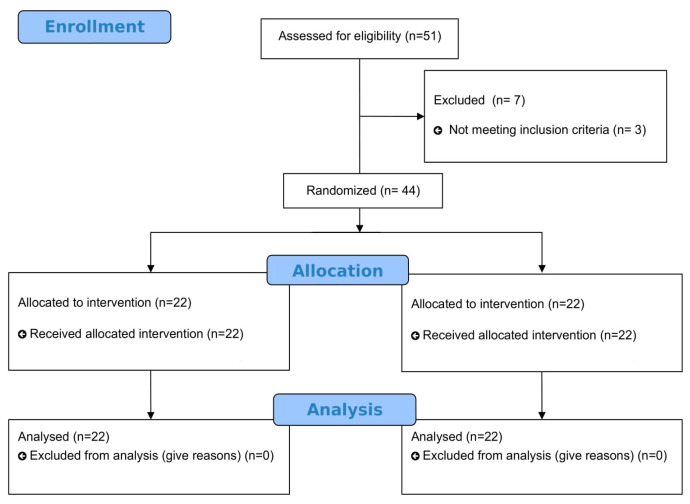
Design and flow chart of the study.

Data were systematically gathered from the medical records of patients diagnosed with cervical disc herniation at the Department of Physical Therapy and Rehabilitation, Istanbul Medipol University, over the period from May 2023 to February 2024. These patients were diagnosed with CDH by a neurologist after neurological examination, clinical physical examination, and radiographic evaluation. The inclusion criteria for the study comprised individuals who had been diagnosed with CDH, had experienced neck pain with a VAS resting score of at least 6 in the last 3 weeks, and exhibited a restricted range of motion below normal values in their cervical joints. The exclusion criteria included individuals with concurrent orthopedic or neurological disorders in addition to cervical issues, those with a history of trauma in the neck area, participants who had recently been involved in a physiotherapy program within the past six months, and those who were unable to tolerate VT (short effects; headache, dizziness, nausea, acute back pain) [30]. Fifty-one participants were screened, and forty-four participants who met the inclusion criteria were included in the study. Three individuals who did not meet the inclusion criteria and four individuals who declined to participate were excluded from the study. The flow chart of the study is presented in Figure 1. 

Forty-four individuals were included in the study and were randomized into two groups. Randomization was performed by placing 44 pieces of paper, half with odd numbers and half with even numbers, into a closed box. A random paper was drawn for each participant included in the study. Participants who drew an odd number were placed in the VG, and participants who drew an even number were placed in the CG.

### 2.2. Intervention

All participants were randomly divided into two groups: the conventional group and the vibration group. In this setup, one of the physiotherapists, who was not involved in the main research, was responsible for conducting both the evaluation and the treatment procedures. This approach ensured that the participants were unaware of the specific treatment group to which they were assigned, maintaining the integrity of the single-blind design. By having a separate physiotherapist handle the evaluations and treatments, the principal investigators could remain blinded to the participants’ group assignments, thereby reducing the potential for bias and enhancing the reliability of the study’s findings.

### 2.3. Conventional Group

The participants in this group received a conventional therapy program four times per week for three weeks. This program included 20 min of heat application to the neck region and Transcutaneous Electrical Nerve Stimulation (TENS) at 100 Hz applied to the painful neck areas. Additionally, Active Range of Motion (AROM) exercises were administered under the supervision of a physiotherapist. These AROM exercises were performed in neck flexion, extension right and left lateral flexion, holding at the end range for 2 s, across 2 sets of 10 repetitions each. Strengthening exercises included isometric exercises in flexion, extension, and right and left lateral flexion, holding at the end range for 6 s. These isometric exercises were conducted over 2 sets of 10 repetitions with a rest period of 1 min between sets. 

### 2.4. Vibration Group

Participants in this group received VT in addition to conventional methods. This therapy was administered using a percussion massage gun (Compex Fix 2.0, Compex; Geneva, Switzerland), applied along the origin-insertion lines of the trapezius, levator scapulae, and cervical paravertebral muscles at medium speed (level 2, range: 33–40 Hz) and 12 mm amplitude [31]. Each muscle group was treated for three minutes. The VT was performed using the soft head attachment of the percussion massage gun. The vibration was applied to the neck region three times a week for four weeks.

### 2.5. Outcome Measurements

The outcome measurements of all participants were made before the treatment and after 3 weeks of treatment.

#### 2.5.1. Laser Pointer Assisted Angle Repetition Test (LI-YATT)

The test was used to evaluate the joint position sense. It was administered following the protocol established by Revel et al. [32]. This test measures joint position sense, a component of proprioception, by providing feedback on the speed and direction of active and passive movements without visual cues. The patient was seated such that there was a distance of 100 cm between them and a target board, which measured 90 × 80 cm. A laser pointer was attached to the patient’s head using an appropriate strap, and positioned so that the laser directly targeted the exact center of the board. Initially, the patient was instructed to target the center of the board (origin) with the laser while looking, then bring their head into flexion. Subsequently, the patient was asked to return their head to a neutral position and target the center again. The patient then had to target the center, close their eyes, and repeat the process, stopping when they felt they were back at the center point. The distance of this point from the origin was then measured [33].

#### 2.5.2. Visual Analog Scale (VAS)

The VAS was used to evaluate the pain intensity of patients. This test comprises a horizontal line measuring 10 cm in length, where ‘0’ denotes the absence of pain, while ‘10’ signifies unbearable pain. During our evaluation, patients were instructed to mark their perceived level of pain on this scale, and the distance from the starting point was measured and recorded using a ruler [34].

#### 2.5.3. Tampa Scale for Kinesiophobia (TSK)

The kinesiophobia of the patients was evaluated. This scale, developed by Tunca et al. in 2011 for the evaluation of patients’ fear of movement, consists of 17 items with established Turkish validity and reliability. Scored on a 4-point Likert scale, with responses ranging from “strongly disagree” 1, “disagree” 2, “agree” 3, to “strongly agree” 4, the scoring of this scale involves reversing the 4th, 8th, 12th, and 16th items before calculating a total score. An individual receives a total score between 17 and 68, with higher scores indicating greater levels of kinesiophobia [35].

#### 2.5.4. Neck Disability Index (NDI)

The NDI was used to evaluate cervical disorder. This scale is designed to gauge both the intensity of pain and the restrictions in daily activities resulting from cervical pathologies. Comprising 10 sections, encompassing aspects such as pain severity, lifting capabilities, reading proficiency, headaches, personal grooming, professional duties, concentration levels, quality of sleep, engagement in recreational activities, and driving competence, each section offers a spectrum of 6 responses, ranging from 0 to 5 points. A score of 0 indicates an absence of pain or functional impediment, while a score of 5 signifies the highest degree of pain and restriction [36].

### 2.6. Statistical Analysis

The study’s required sample size was estimated to be 42 with 80% power (α = 0.05), and effect size (d = 0.80) according to the VAS-resting value using the G*power sample size (3.1.9.2) calculator [37]. Statistical Package for Social Sciences (SPSS) version 22.0 was used for statistical analysis. The descriptive statistics of the variables such as age, height, weight, and all measurements before and after treatment were shown as mean ± SD. A Wilcoxon test was used for intra-group comparisons before and after treatment. The effect size was calculated using Cohen’s d. Effect sizes were interpreted according to Cohen’s criteria (small ≤ 0.2; moderate = 0.5; large ≥ 0.8) [38]. Associated 95% confidence intervals (CI) estimated reliability. The significance value was accepted as *p* < 0.05.

## 3. Results

Each treatment group consisted of 22 individuals who were diagnosed with CDH, which is chronic neck pain lasting more than 6 weeks, a total of 44 participants completed the study. When examining the demographic information of the individuals participating in the study, the parameters of age, weight, height, dominant side, and affected side were similar across both groups. The demographic and physical information of the participants is presented in Table 1. Upon examining the pre-treatment evaluation parameters of the groups, VAS resting, VAS activity, TSK, NDI, and all parameters of joint position sense (JPS) were found to be similar across both groups. (*p* > 0.05). When the pre-treatment and post-treatment values of the VG group were examined, a significant improvement was observed in all parameters except neck left rotation (*p* < 0.05). A significant improvement was observed in the pre- and post-treatment values of all parameters of the CG group (*p* < 0.05) (Table 2). When a comparison was made between the groups, the values of VAS resting, TSK, NDI, JPS neck flexion, and JPS neck extension were similar in both groups (*p* > 0.05). When comparing the difference values between groups, the VG was found to be more effective than the CG in the parameters of VAS activity (*p* = 0.013). The CG had more improvement in JPS neck left rotation than the VG (*p* = 0.000) (Table 2).

## 4. Discussion

The main finding of the study was that improvements in pain, kinesiophobia, disability, and some proprioception parameters were observed in all groups before and after therapy. In the discussion of our findings, it was observed that the vibration group showed greater efficacy during activity-related pain. Conversely, the conventional group demonstrated higher effectiveness in the JPS parameter for left rotation. The results for the other parameters were similar between the two groups.

Cervical pain is a multifactorial disease and a major problem in modern societies. Even though neck pain is not the most common musculoskeletal disorder, it is still a very important one [39]. Although many treatment options can be used in CDH, there are fewer studies in the literature compared to the lumbar region. Neck vibration methods, regardless of the specific modality, may be effective for treating pain and disability in patients with chronic neck pain [9]. Neck muscle vibration shows different effects on neck pain patients and healthy controls. VT may be an effective intervention for reducing self-reported neck pain, disability, and pressure pain sensitivity in patients with chronic, non-specific neck pain and could be recommended for these individuals [9,40]. Beinert et al. found that neck muscle vibration had short-term general effects on analgesia and sensorimotor function, with longer-term specific effects. They also suggested that future studies should investigate the potential benefits of neck muscle vibration as an adjunct to physical therapy to improve cervical sensorimotor function. They concluded that a single session of neck muscle vibration improved cervical joint position sense and arm-matching acuity in neck pain patients for up to 24 h. Additionally, they observed that neck muscle vibration increased pressure pain thresholds in the stimulated region while decreasing them in the distal region [11]. Furthermore, another study showed that 10 sessions of using 35–50 Hz frequency ranges improved pressure pain sensitivity over the trapezius and levator scapulae and self-reported neck pain in patients with chronic non-specific neck pain [9]. When we analyzed our findings, a significant improvement in the pain parameter in all treatment groups was found in parallel with the literature. In addition, better results were obtained in activity-related pain in the neck VG compared to the CG. It is believed that local vibration therapy using a massage gun increases arterial blood flow and reduces pain, but this has yet to be proven [41]. This study showed that local vibration therapy applied in the 33–40 Hz range reduced neck pain in patients with CDH.

As far as we know, there are very few studies examining the effect of VT on kinesiophobia in CDH in the literature. It has been seen that studies are mostly related to kinesiophobia and neck pain. In a recent study, 87 non-specific CNP individuals demonstrated no interaction between neck pain intensity and kinesiophobia [42]. It has been stated that the fear of movement increases due to pain and discomfort in patients with chronic neck pain. In an exploratory analysis, Vaegter et al. showed that people with chronic musculoskeletal pain had greater levels of kinesiophobia and more intense pain, and vice versa [43]. Also, in one study, participants received conventional treatment methods, including heat application, TENS, and range of motion exercises, supplemented by VT. The study found that adding VT to the treatment regimen improved pain levels, functionality, and proprioception in patients with CDH. It has been emphasized that patients whose proprioception improved also experienced a reduction in kinesiophobia in individuals with CDH [44]. In our study, in patients with CDH to whom we applied VT, it was similar to the group in which we applied conventional treatment, and it improved kinesiophobia.

In our study, both treatment methods provided similar improvements in NDI scores, indicating that both methods have the potential to improve patients’ daily living activities. In this study, it was found that NDI scores showed significant improvements in both groups before and after treatment. This demonstrates that both vibration therapy and conventional therapy are effective in enhancing functionality in individuals with CDH. However, no significant difference was found between the groups. This finding indicates that both treatment methods are equally effective. Cleland et al. reported that a combination of manual therapy and exercise significantly improved NDI scores in chronic neck pain [45]. In a study examining the effects of VT on NDI scores in individuals with neck pain, it was found that there was a 44% improvement in NDI scores [9]. We believe that the improvement in NDI scores in both groups was due to the conducting of conservative treatment in both groups in our study.

Conventional treatment methods typically include various modalities such as manual therapy, and exercise. These methods improve functionality by reducing muscle tension, increasing the range of motion, and alleviating pain. Previous studies also report that conventional physiotherapy and VT are effective in enhancing functionality [9,46]. In a study by Dueñas et al., it was reported that low-frequency self-applied VT provided a significant improvement in NDI scores in patients with chronic non-specific neck pain [9]. Studies with larger sample sizes and long-term follow-ups are needed to further validate these findings and to elucidate the long-term effects of vibration therapy. One meta-analysis study determined that the relationship between pain, and disability is interconnected. It is possible to observe improvement in disability scores due to the pain-reducing effect of vibration [47].

A significant issue faced by patients suffering from neck pain is the disruption of cervical proprioception. Recent research highlights that disturbances in cervical sensorimotor control often stem from this proprioceptive dysfunction. Numerous tests have been developed to evaluate this aspect in individuals with chronic neck pain, of which the Joint Position Sense test is the most frequently utilized [3,48]. In the systematic review conducted by Vriev and colleagues, it was indicated that participants with neck pain exhibited a significantly higher Joint Position Sense Error compared to healthy controls [49]. Consistent with the literature, we used the JPS test to evaluate proprioception in the participants with CDH. According to our findings, there were significant differences in JPS neck measurements in both groups. Only a more significant improvement was seen in the JPS neck left rotation value of the CG group compared to the VG. The reason for the lack of more difference in changes in the VG than CG may be that the treatment sessions were not long enough to change neck JPS.

In a study examining the effect of neck vibration on immediate joint position sense, it was reported that a 5 min vibration application instantaneously improved joint position sense [50]. In a study conducted by Beinert and colleagues, it was reported that VT applied to the neck region in individuals with neck pain was effective in improving joint position sense [10]. Another study reported that short-term VT applied to the neck region could temporarily improve joint position sense. This study emphasized that there is limited research on vibration applications for neck pain in the literature, and there is a need for more comprehensive studies [11]. Although the literature reports that VT improves neck proprioception, studies in this area are limited. Also, some of these studies examining the effects of VT applied to the neck on joint position sense were conducted on patients with non-specific neck pain. Examining the effects of VT on CDH is one of the strengths of our study. In our study, both groups, those receiving conventional exercise and those combining conventional exercise with VT, showed improvements in JPS of the neck. We hypothesize that exercise applications reduce pain, which in turn could enhance proprioception, and that vibration applications may stimulate the proprioceptive system, contributing to joint position sense.

In the study conducted by Lee and colleagues, it was reported that a 5-week cervical stabilization exercise training improved joint position sense [51]. Another study reported that in individuals with chronic neck pain, specific neck exercises and general exercises for the neck and shoulder girdle were effective in enhancing proprioception [52]. Fatima and colleagues have recommended the combined use of stabilization and proprioceptive exercises to improve proprioception and reduce pain in individuals with neck pain [53]. The literature reports that stabilization and strengthening exercise approaches applied to the neck area have been shown to improve joint position sense. Similarly to the literature, the four-week conventional exercise program implemented in our study contributed to enhancing the neck JPS.

The inclusion of many parameters of the vibration treatment is the study’s primary strength. The limited number of studies examining the effect of VT in participants diagnosed with CDH increases the strength of our study.

One of the limitations of our study is that the long-term effects of the interventions were not examined. The small number of participants and the lack of use of computerized evaluation systems were additional limitations. Another limitation is that we did not determine the disc herniation levels of the participants diagnosed with CDH. We recommend that future studies should be conducted on participants with a specific stage of disc herniation. Also, future studies should further explore the effect of VT in chronic pain problems of other body regions.

Future research should focus on larger sample sizes, longer follow-up periods, and exploring different frequencies and durations of VT to further validate these results and to better understand the long-term effects and optimal parameters of VT. Additionally, we consider that local VT applications applied at different frequencies and durations are needed in the future to investigate the standardized frequency and duration in which VT increases proprioception and reduces pain in CDH. Future research should develop a standard, verified treatment regimen to reduce pain and improve proprioception.

## 5. Conclusions

This study demonstrated that both conventional physiotherapy and VT are effective in improving pain, kinesiophobia, disability, and proprioception in individuals with CDH. A significant improvement was observed in all parameters except neck left rotation in comparison to pre- and post-treatment values in VG. While the VG exhibited superior results in reducing activity-related pain than CG, the CG showed better outcomes in joint position sense for left rotation than VG. The findings suggest that VT can be a valuable adjunct to conventional physiotherapy for managing CDH, enhancing pain relief, and improving proprioception, kinesiophobia, and disability. Given the safety and potential benefits of VT, incorporating it into rehabilitation programs for patients with CDH is recommended. We suggest that local VT is an alternative to conventional physiotherapy that can be used to reduce the symptoms of CDH. The results of this investigation may help practitioners decide which applications to use or not.

## Figures and Tables

**Table 1:** Distribution of the demographic and physical information.

	Vibration Group (Mean ± SD)	Conventional Group (Mean ± SD)	*p*-Value
Age (years)	44.40 ± 2.97	45.45 ± 2.70	0.338
Weight (kg)	71.45 ± 7.04	70.40 ± 6.67	0.721
Height (cm)	167.13 ± 8.86	170.45 ± 11.22	0.139
Sex (F/M)	9/13	8/14	0.235
Dominant side			
Right	19 (86.4)	15 (68.2)	0.567
Left	3 (13.6)	7 (31.8)	0.356
Affected side			
Right	17 (77.2)	14 (63.6)	0.431
Left	5 (22.8)	8 (36.4)	0.134

SD: Standard deviation, kg: kilogram, cm: centimeter.

**Table 2:** Comparison of changes in outcome measures within and between the groups.

Variables	Vibration Group			Cohen d	Conventional Group			Cohen d	Diff (95% CI) Upper/Lower	Effect Size
	Pre-Treatment	Post-Treatment	*p*		Pre-Treatment	Post-Treatment	*p*			
VAS/resting	6.47 ± 1.96	2.78 ± 1.56	0.00	2.08	7.30 ± 1.42	4.90 ± 0.57	0.00	2.21	−2.42/−0.13	1.30
VAS/activity	7.01 ± 1.15	3.07 ± 1.77	0.00	0.64	7.49 ± 0.76	4.99 ± 1.07	0.00	2.69	−2.41/−0.47	0.81
TSK	47.77 ± 10.45	27.50 ± 6.08	0.00	2.37	50.68 ± 8.95	41.09 ± 8.47	0.00	1.10	−16.61/−4.75	1.55
NDI	25.00 ± 6.62	12.77 ± 2.77	0.00	2.41	20.27 ± 5.16	13.40 ± 2.42	0.00	2.96	−9.08/−1.64	1.03
JPS-neck flexion	7.80 ± 0.97	4.05 ± 0.92	0.00	3.96	7.28 ± 0.89	6.01 ± 0.75	0.00	1.54	−3.17/−1.80	2.88
JPS-neck ext.	8.13 ± 1.35	5.11 ± 1.07	0.00	2.47	8.23 ± 1.18	7.08 ± 0.57	0.00	1.24	−2.71/−1.05	2.20
JPS-neck right rot.	7.24 ± 1.52	6.03 ± 1.22	0.03	0.87	7.55 ± 1.08	6.67 ± 0.92	0.01	0.87	−1.21/0.52	0.35
JPS-neck left rot.	6.34 ± 1.43	5.35 ± 1.38	0.05	0.70	8.25 ± 1.02	6.75 ± 0.83	0.00	1.61	−0.61/1.62	0.30

VAS: Visual analog scale, TSK: Tampa Scale for Kinesiophobia, NDI: Neck Disability Index, JPS: Joint position sense, rot: rotation, diff: difference, ext: extension.

## Data Availability

The dataset analyzed during the study is not publicly available according to the study protocol. However, de-identified data may be obtained from the corresponding author with permission from Istanbul Medipol University upon reasonable request.
